# Associations Between Hospice Care Duties and Compassion Fatigue Among Nurses in Korea: The Roles of End‐of‐Life Care Stress and Peer Support

**DOI:** 10.1155/jonm/4850869

**Published:** 2026-11-01

**Authors:** Haerim Lee, Hyun-E Yeom

**Affiliations:** ^1^ Department of Nursing Chungnam National University 266, Munhwa-ro, Jung-gu Dae-jeon South Korea cnu.ac.kr

**Keywords:** compassion fatigue, hospice care, peer influence, social support, stress, terminal care, workload

## Abstract

**Aim:**

To examine associations between nurses’ appraisals of hospice care duties and compassion fatigue and the roles of end‐of‐life care stress (EOLCS) and peer support in these associations among hospice nurses in Korea.

**Background:**

Compassion fatigue is relevant to hospice nurses’ well‐being and care quality, yet its associations with duty appraisals, EOLCS, and peer support remain insufficiently understood.

**Methods:**

This cross‐sectional study analyzed survey data from 117 nurses recruited through 12 specialized inpatient hospice units in Korea. Compassion fatigue was operationalized using the secondary traumatic stress subscale of the Professional Quality of Life Scale. Cross‐sectional indirect associations and interactions were examined using the PROCESS macro for SPSS. Reporting followed the STROBE guidelines.

**Results:**

Among nurses’ appraisals of hospice care duties, perceived duty difficulty showed a positive cross‐sectional indirect association with compassion fatigue through EOLCS (effect = 2.82, 95% bootstrap CI [1.08, 4.73]). Its interaction with peer support was significant (*B* = −6.12, *p* = 0.009), showing a crossover pattern. The simple slope was nonsignificant at low peer support, and only 3.4% of participants fell within the significant positive region identified by the Johnson–Neyman analysis. At high peer support, duty difficulty was negatively associated with compassion fatigue. Corresponding indirect associations and interactions involving duty importance and frequency were nonsignificant.

**Conclusion:**

Perceived duty difficulty showed a cross‐sectional indirect association with compassion fatigue through EOLCS, while its conditional association varied across peer support levels. These findings highlight the relevance of considering perceived duty difficulty together with EOLCS and peer support when examining compassion fatigue among hospice nurses.

**Implications for Nursing Management:**

Structured peer support, debriefing, mentoring, and monitoring approaches addressing difficult care duties and psychological distress warrant prospective development and evaluation.

## 1. Introduction

Hospice care is a comprehensive, patient‐centered approach that enhances quality of life for individuals with life‐limiting illnesses and their families by integrating physical, psychosocial, and spiritual care through interprofessional collaboration [1]. In Korea, national health insurance coverage for inpatient hospice services is exclusively available to patients with terminal‐stage cancer [2]. Hospice nurses therefore primarily provide direct bedside care and care coordination for terminally ill cancer patients and their families, with clinical responsibilities that encompass end‐of‐life symptom management, facilitation of advance care planning, and family‐centered psychosocial support.

Compassion, conceptualized as recognizing another’s suffering and responding through empathic engagement, is regarded as a core value of nursing practice [3]. In hospice settings, nurses encounter profound emotional demands, including patients’ existential distress as they confront mortality, grief, and complex family dynamics. Prolonged empathic engagement and repeated exposure to such suffering place nurses at heightened risk of compassion fatigue, a state of emotional and psychological exhaustion [4, 5]. Compassion fatigue undermines nurses’ quality of life, job satisfaction, and professional identity, making its prevention and management essential for sustaining high‐quality care and work‐related well‐being [6, 7].

Beyond exposure to suffering itself, understanding compassion fatigue in hospice nurses requires attention to how nurses perceive and appraise the demands of their care duties. Such appraisals may shape their work experiences and professional quality of life [8–10]. Empirical evidence from diverse clinical settings indicates that appraisals of duty importance, frequency, and difficulty are associated with key work‐related outcomes, including job satisfaction, work engagement, and burnout [6, 7, 9]. In hospice care, these appraisals are embedded within multidisciplinary activities spanning physical, psychosocial, and spiritual domains. However, how hospice nurses appraise their specific inpatient care duties and how these appraisals relate to compassion fatigue remain inadequately understood.

End‐of‐life care entails substantial practical demands and psychological burden. Nurses providing such care frequently experience significant end‐of‐life care stress (EOLCS), characterized by emotional exhaustion from managing complex symptoms, preserving patients’ dignity, and preventing avoidable suffering [11, 12]. This stress is compounded by ethically challenging situations such as withholding or withdrawing life‐sustaining treatment and mediating conflicts among patients, families, and physicians [13, 14]. Prior evidence indicates that EOLCS is closely associated with work‐related conditions such as high workload, staff shortages, and time pressure [11, 12]. Moreover, repeated exposure to dying patients and grieving families intensifies feelings of worthlessness, isolation, and frustration that overlap with compassion fatigue [4, 5, 15–20]. These findings suggest that EOLCS may function as a key psychological pathway linking nurses’ appraisals of their care duties to compassion fatigue.

Peer support refers to the emotional, informational, and practical assistance that colleagues provide to one another based on shared professional experiences [21]. It encompasses empathic listening, sharing of clinical experiences, and mutual encouragement in the face of work‐related challenges and is recognized as a critical contextual resource for sustaining nurses’ well‐being and professional functioning [21]. Peer support may be particularly crucial in inpatient hospice units, where nurses routinely face high‐intensity end‐of‐life care demands, ethically complex decision‐making, and ongoing support for grieving families, all of which contribute to psychological exhaustion. Evidence indicates that peer support enhances job satisfaction and buffers work‐related distress by attenuating the negative impact of traumatic work events [22, 23], thereby helping nurses maintain empathic engagement. In contrast, insufficient peer support can heighten frustration, isolation, and perceived workload burden, thereby increasing compassion fatigue risk [5, 7, 24, 25]. However, in hospice settings where nurses’ duties involve highly specialized and emotionally charged care, the extent to which peer support shapes the relationship between nurses’ appraisals of their hospice care duties and compassion fatigue remains insufficiently understood.

Given the implications of compassion fatigue for both hospice nurses’ well‐being and the quality of patient care, identifying potentially modifiable factors within hospice settings is important. Prior research has highlighted emotional demands, including EOLCS, and work‐related contextual factors, such as perceived workload, time pressure, and available support as relevant to compassion fatigue [4–7, 11–14]. However, how nurses’ appraisals of hospice care duties relate to compassion fatigue in the context of EOLCS and peer support remains insufficiently understood. Building on this literature, the present study examined associations between nurses’ appraisals of hospice care duties—importance, frequency, and difficulty—and compassion fatigue among those working in Korean inpatient hospice units. Specifically, it examined cross‐sectional indirect associations through EOLCS and whether the associations between duty appraisals and compassion fatigue varied according to peer support.

Figure 1 presents the hypothesized framework for this study. Duty appraisals were hypothesized to be associated with EOLCS, and higher EOLCS was expected to be associated with greater compassion fatigue. The associations between duty appraisals and compassion fatigue were also expected to vary according to peer support.

**FIGURE 1:**
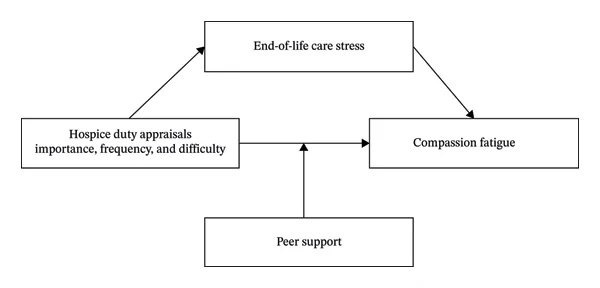
Conceptual framework of the associations between hospice duty appraisals and compassion fatigue, including indirect associations through end‐of‐life care stress and interactions with peer support.

## 2. Methods

### 2.1. Sample and Procedure

This study used a descriptive, cross‐sectional design. The target population comprised registered nurses working in inpatient hospice units. In accordance with Korean healthcare policies governing inpatient hospice care, participants were recruited from 12 hospitals located in three regions of South Korea (Daejeon, Chungcheong, and Jeolla). All participating hospitals were designated as specialized hospice facilities and operated under unified national hospice care policies. Data were collected between January 13 and February 14, 2020.

Eligibility criteria included a minimum of 6 months’ employment in an inpatient hospice unit and primary responsibility for direct patient care rather than managerial duties. These criteria were intended to ensure sufficient exposure to intensive end‐of‐life care demands and ongoing patient‐family interactions associated with compassion fatigue development. Nurses who had experienced the bereavement of a close relative within the preceding 6 months were excluded to minimize potential confounding between acute personal grief and work‐related stress or compassion fatigue.

Following ethical approval from the university’s institutional review board, data were collected via an online survey using convenience sampling. Recruitment notices outlining the study objectives, procedures, and eligibility criteria were posted in inpatient hospice units. Interested nurses accessed the survey by scanning a QR code, provided electronic informed consent, and completed the questionnaire.

Of 121 nurses who initially provided informed consent, four did not complete the survey, yielding 117 completed responses. No item‐level missing data were identified among the completed questionnaires included in the final analyses. A sensitivity power analysis using G∗Power 3.1.9.7 indicated a minimum detectable interaction effect of *f* ^2^ = 0.091 with 90% power at *α* = 0.05 for the final sample.

### 2.2. Measures

#### 2.2.1. Compassion Fatigue

The Professional Quality of Life Scale (ProQOL) is an instrument developed to assess professional quality of life among professionals who help others in diverse social contexts, including healthcare settings, and who perform emotional labor and/or are exposed to traumatic situations [5]. It comprises three subscales—Compassion Satisfaction, Burnout, and Secondary Traumatic Stress—with no subscale formally labeled “compassion fatigue.” Following Figley’s original conceptualization, in which compassion fatigue is understood as equivalent to secondary traumatic stress [26], the secondary traumatic stress subscale was used to operationalize compassion fatigue in this study. This subscale consists of 10 items rated on a 5‐point Likert scale from 1 (*never*) to 5 (*very often*), and a summed score (range 10–50) was calculated, with higher scores indicating greater compassion fatigue. The internal consistency in this study was Cronbach’s *α* = 0.81. The ProQOL [5] is an open‐access instrument available for noncommercial use.

#### 2.2.2. Appraisals of Hospice Care Duties

Nurses’ perceptions of care duties in inpatient hospice units were assessed using the Task Questionnaire, which was developed and validated for hospice nurses in Korea [9]. The questionnaire comprises 37 items covering eight domains of duties that nurses routinely perform in inpatient hospice settings. Each item is rated on three 4‐point Likert‐type subscales assessing perceived importance, frequency, and difficulty. The subscale for importance evaluates how significant each duty is perceived to be, the frequency subscale assesses how often nurses perform each duty, and the difficulty subscale reflects how challenging nurses perceive each duty to be. The three subscale scores were computed as item means, with higher scores indicating greater importance, more frequent performance, and higher perceived difficulty. Internal consistency (Cronbach’s *α*) for importance, frequency, and difficulty in the present study was 0.97, 0.98, and 0.98, respectively. The scale was used in this study with permission from the original developer [9].

#### 2.2.3. EOLCS

Nurses’ stress related to providing end‐of‐life care for terminally ill patients was assessed with the End‐of‐Life Care Stress Scale [27]. The scale comprises 40 items assessing the extent to which nurses caring for terminally ill patients experience stress. Respondents rate the level of stress on a 5‐point Likert scale from 1 (*not at all*) to 5 (*very true*). A mean score was calculated across all items, with higher scores indicating greater stress. Internal consistency in this study was Cronbach’s *α* = 0.93. Use of the scale was authorized by Lee [27], the developer of the original instrument.

#### 2.2.4. Peer Support

Peer support was assessed using the Social Provision Scale (SPS), originally developed by Cutrona and Russell [28] and validated for use with Korean nurses [29]. The SPS consists of 20 items that assess the extent to which respondents perceive receiving support from colleagues in their clinical practice. Each item is rated on a 5‐point Likert scale ranging from 1 (*not at all true*) to 5 (*extremely true*). A mean score was calculated across all items, with higher scores indicating stronger perceived peer support. Internal consistency in this study was Cronbach’s *α* = 0.94. Use of the scale was authorized by the original developer [28].

### 2.3. Statistical Analysis

Data analyses were conducted using SPSS Version 29.0 (IBM Corp., Armonk, NY, USA). The characteristics of the participants and main study variables (i.e., appraisals of the importance, frequency, and difficulty of hospice care duties; EOLCS; peer support; and compassion fatigue) were summarized with descriptive statistics (i.e., frequencies, percentages, means, and standard deviations). Bivariate relationships were examined using Pearson correlation coefficients and independent *t*‐tests. Potential covariates were identified based on prior literature and their conceptual relevance to the study variables. Previous studies have identified educational level and clinical experience as potentially relevant factors in compassion fatigue among nurses [7, 11, 30]. In the present sample, education level was also significantly associated with perceived duty importance and frequency, whereas hospice work experience was significantly associated with EOLCS. Accordingly, education level and hospice work experience were included as covariates.

The hypothesized model shown in Figure 1 was tested using the PROCESS macro (Model 5) for SPSS. Three separate PROCESS Model 5 analyses were conducted for perceived duty importance, frequency, and difficulty, with the remaining duty appraisal variables included as covariates in each model. All regression coefficients (B) reported in this study are unstandardized. Education was coded as a binary variable (0 = bachelor’s degree or below, 1 = master’s degree or above). Variance inflation factors ranged from 1.26 to 1.71, indicating no problematic multicollinearity. Continuous predictor and moderator variables involved in the interaction terms were mean‐centered prior to constructing the product terms. Heteroscedasticity‐consistent standard errors were estimated using HC3.

Cross‐sectional indirect associations between the three duty‐appraisal dimensions and compassion fatigue through EOLCS were estimated. These associations were interpreted as exploratory statistical associations rather than evidence of temporal ordering or causal mediation. Interactions between peer support and each duty‐appraisal dimension were examined in relation to compassion fatigue by including the corresponding product term in each outcome model. Significant interactions were further probed using simple‐slope analyses at the mean and ±1 SD of peer support and the Johnson–Neyman technique. Regression coefficients and conditional associations were evaluated using HC3‐based inference, whereas cross‐sectional indirect associations were evaluated using 95% bootstrap confidence intervals based on 5000 resamples and were considered statistically significant when the interval did not include zero.

To assess the robustness of both the cross‐sectional indirect associations through EOLCS and the interactions with peer support, each of the three models was re‐estimated with its focal appraisal retained and the other two appraisal dimensions excluded. Education level and hospice work experience remained included as covariates in all models. The same bootstrap and HC3 procedures used in the primary analyses were applied.

A sensitivity power analysis was conducted using G∗Power to determine the minimum detectable interaction effect with 117 participants, *α* = 0.05, and 90% power. The calculation specified eight predictor terms in the outcome model and evaluated sensitivity to the increase in *R* ^2^ attributable to a single interaction term. It assumed conventional multiple regression conditions and did not establish power for the cross‐sectional indirect associations.

### 2.4. Ethical Considerations

This study was approved by the institutional review board of the affiliated institution (approval no. 201906‐SB‐095‐01). In accordance with the Declaration of Helsinki, all procedures were designed to safeguard participants’ rights, dignity, and well‐being. Participation was voluntary, and electronic informed consent was obtained after a standardized explanation of the study purpose, procedures, potential risks and benefits, use of data, and contact information for the research team. Participants were informed that they could decline participation, withdraw at any time, or skip any item without penalty and, given the sensitivity of hospice nursing, could pause or terminate the survey if they experienced discomfort. Privacy and confidentiality were protected by assigning anonymous study identifiers, removing personally identifiable information, and storing de‐identified data on password‐protected servers with access restricted to the research team.

## 3. Results

### 3.1. Sociodemographic Characteristics of Participants

Most participants were women (95.7%), with a mean age of 37.93 years (SD = 10.71). The majority had an education level below a master’s degree (78.6%) and were staff nurses (80.3%); the remaining participants were charge nurses, who also provided direct patient care in the hospice unit. Participants had worked an average of 41.91 months in the hospice unit (SD = 41.60, median = 36.00, range = 6–180 months), and 79.5% of the participants had received specialized hospice‐related continuing education within the past year (Table 1). Table 1 also presents the associations of sociodemographic characteristics with compassion fatigue. None of the sociodemographic characteristics were significantly associated with compassion fatigue.

**TABLE 1:** Participant characteristics and their associations with peer support and compassion fatigue (*N* = 117).

Characteristics	Categories	*n* (%) or M ± SD	Peer support		Compassion fatigue	
			M (SD)	*t* or *r* (*p*)	M (SD)	*t* or *r* (*p*)
Gender	Male	5 (4.3)	3.63 (0.50)	−0.66 (0.510)	24.80 (4.15)	−1.00 (0.320)
	Female	112 (95.7)	3.81 (0.62)		27.49 (5.94)	

Age (years)		37.93 ± 10.71 (range = 23–68)		−0.04 (0.670)		−0.01 (0.938)
Marital status	Married	62 (53.0)	3.85 (0.61)	0.82 (0.416)	27.13 (6.58)	−0.48 (0.632)
	Single or others	55 (47.0)	3.76 (0.62)		27.65 (5.05)	

Religion	Yes	64 (54.7)	3.87 (0.60)	1.28 (0.205)	27.52 (5.77)	0.28 (0.780)
	No	53 (45.3)	3.73 (0.62)		27.21 (6.09)	

Education	Bachelor’s degree or below	92 (78.6)	3.77 (0.60)	−1.25 (0.215)	27.50 (5.72)	0.44 (0.664)
	Master’s degree or above	25 (21.4)	3.94 (0.65)		26.92 (6.60)	

Position	Staff nurse	94 (80.3)	3.77 (0.61)	−1.27 (0.206)	27.45 (5.97)	0.26 (0.794)
	Charge nurse	23 (19.7)	3.95 (0.62)		27.09 (5.69)	

Work schedule	Shift work	87 (74.4)	3.77 (0.60)	−1.03 (0.304)	27.30 (5.62)	−0.24 (0.810)
	Fixed day shift	30 (25.6)	3.91 (0.65)		27.60 (6.71)	

Hospice work experience (months)		41.91 ± 41.60 (range = 6–180)		0.32 (< 0.001)		0.09 (0.313)
Hospice continuing education in the past year	Yes	93 (79.5)	3.86 (0.63)	1.92 (0.057)	27.20 (6.37)	−0.86 (0.392)
	No	24 (20.5)	3.60 (0.48)		28.04 (3.50)	

### 3.2. Relationships Among Main Study Variables

Table 2 presents the descriptive statistics of the main study variables and their bivariate correlations. Mean scores were as follows: appraisals of hospice care duties—importance (*M* = 3.49, SD = 0.41), frequency (*M* = 2.96, SD = 0.48), difficulty (*M* = 2.87, SD = 0.47)—EOLCS (*M* = 3.89, SD = 0.54), peer support (*M* = 3.81, SD = 0.61), and compassion fatigue (*M* = 27.38, SD = 5.89).

**TABLE 2:** Relationships among hospice duty appraisals, end‐of‐life care stress, peer support, and compassion fatigue (*N* = 117).

Variables	M (SD)	1‐1	1‐2	1‐3	2	3	4
		*r* (*p*)	*r* (*p*)	*r* (*p*)	*r* (*p*)	*r* (*p*)	*r* (*p*)
1. Hospice duty appraisals							
1‐1. Perceived duty importance	3.49 (0.41)	—					
1‐2. Perceived duty frequency	2.96 (0.48)	0.57 (< 0.001)	—				
1‐3. Perceived duty difficulty	2.87 (0.47)	−0.05 (0.618)	−0.13 (0.162)	—			
2. End‐of‐life care stress	3.89 (0.54)	0.13 (0.157)	0.05 (0.605)	0.43 (< 0.001)	—		
3. Peer support	3.81 (0.61)	0.32 (< 0.001)	0.20 (0.035)	−0.16 (0.083)	−0.04 (0.673)	—	
4. Compassion fatigue	27.38 (5.89)	0.03 (0.726)	0.04 (0.691)	0.06 (0.554)	0.35 (< 0.001)	−0.06 (0.529)	—

Among the three appraisals of hospice care duties, perceived duty importance (*r* = 0.32, *p* < 0.001) and duty frequency (*r* = 0.20, *p* = 0.035) were positively associated with peer support, whereas perceived duty difficulty was positively correlated with EOLCS (*r* = 0.43, *p* < 0.001). EOLCS was also positively correlated with compassion fatigue (*r* = 0.35, *p* < 0.001).

Table 3 provides details on nurses’ appraisals of hospice care duties in terms of importance, frequency, and difficulty. The duty rated as most important was “quality control and participation in professional development” (*M* = 3.57), followed by “education and counseling” (*M* = 3.54) and “data collection” (*M* = 3.52). The most frequently performed duty was “data collection” (*M* = 3.33), followed by “clinical decision‐making: care planning and evaluation” (*M* = 3.07) and “therapeutic intervention and nursing care” (*M* = 2.98). The duty perceived as most difficult was “education and counseling” (*M* = 2.96), followed by “quality control and participation in professional development” (*M* = 2.90), and both “data collection” and “coping with emergencies” (*M* = 2.89).

**TABLE 3:** Nurses’ hospice duty appraisals: importance, frequency, and difficulty (*N* = 117).

Duties	Importance	Frequency	Difficulty
	M (SD)	M (SD)	M (SD)
Data collection	3.52 (0.39)	3.33 (0.39)	2.89 (0.43)
Clinical decision‐making: care planning and evaluation	3.46 (0.45)	3.07 (0.55)	2.84 (0.53)
Therapeutic intervention and nursing care	3.51 (0.45)	2.98 (0.52)	2.84 (0.58)
Coping with emergencies	3.43 (0.56)	2.81 (0.69)	2.89 (0.60)
Education and counseling	3.54 (0.54)	2.85 (0.75)	2.96 (0.67)
Environment and resource management	3.42 (0.53)	2.65 (0.69)	2.84 (0.65)
Consultation, regulation, and collaboration	3.32 (0.68)	2.47 (0.85)	2.83 (0.78)
Quality control and participation in professional development	3.57 (0.47)	2.84 (0.71)	2.90 (0.66)
Total	3.49 (0.41)	2.96 (0.48)	2.87 (0.47)

### 3.3. Cross‐Sectional Indirect Associations Between Nurses’ Duty Appraisals and Compassion Fatigue Through EOLCS

Table 4 presents cross‐sectional indirect associations between nurses’ appraisals of hospice care duties and compassion fatigue through EOLCS. Each model adjusted for education level, hospice work experience, and the other two duty‐appraisal dimensions. Perceived duty difficulty showed a positive cross‐sectional indirect association with compassion fatigue through EOLCS (effect = 2.82, Boot SE = 0.94, 95% bootstrap CI [1.08, 4.73]). The corresponding cross‐sectional indirect associations for perceived duty importance and frequency were not statistically significant.

**TABLE 4:** Associations of hospice duty appraisals with EOLCS and compassion fatigue (*N* = 117).

Predictor	EOLCS		
	*B* (SE)	*p*	95% CI
Duty importance	0.14 (0.12)	0.261	[−0.10, 0.37]
Duty frequency	−0.02 (0.10)	0.841	[−0.21, 0.17]
Duty difficulty	0.52 (0.13)	< 0.001	[0.26, 0.78]
Model summary: *R* ^2^ = 0.2773; *F*_HC3_ (5, 111) = 10.57, *p* < 0.001			

**Predictor**	**Compassion fatigue**		
	***B* (SE)**	**p**	**95% CI**

Duty importance	−0.02 (1.62)	0.993	[−3.23, 3.20]
EOLCS	4.50 (1.24)	< 0.001	[2.04, 6.96]
Peer support	−0.79 (1.33)	0.551	[−3.42, 1.83]
Duty importance × peer support	−2.33 (2.57)	0.366	[−7.43, 2.76]
*R* ^2^ = 0.1638; *F*_HC3_(8, 108) = 2.99, *p* = 0.005; interaction Δ*R* ^2^ = 0.0085			
Duty frequency	0.31 (1.65)	0.852	[−2.97, 3.59]
EOLCS	4.56 (1.28)	< 0.001	[2.03, 7.09]
Peer support	−0.66 (1.53)	0.669	[−3.69, 2.38]
Duty frequency × peer support	0.57 (2.61)	0.828	[−4.61, 5.75]
*R* ^2^ = 0.1561; *F*_HC3_ (8, 108) = 2.98, *p* = 0.005; interaction Δ*R* ^2^ = 0.0008			
Duty difficulty	−0.54 (1.50)	0.719	[−3.52, 2.43]
EOLCS	5.44 (1.25)	< 0.001	[2.97, 7.91]
Peer support	−0.30 (1.14)	0.792	[−2.56, 1.96]
Duty difficulty × peer support	−6.12 (2.29)	0.009	[−10.65, −1.59]
*R* ^2^ = 0.2374; *F*_HC3_ (8, 108) = 3.75, *p* < 0.001; interaction Δ*R* ^2^ = 0.0821			

**Cross-sectional indirect associations with compassion fatigue through EOLCS**			
	**Indirect estimate**	**Boot SE**	**95% bootstrap CI**

Duty importance	0.61	0.58	[−0.43, 1.86]
Duty frequency	−0.09	0.45	[−1.11, 0.68]
Duty difficulty	2.82	0.94	[1.08, 4.73]

*Note:* EOLCS = end‐of‐life care stress; B = unstandardized coefficient; Boot SE = bootstrap standard error; HC3 = heteroscedasticity‐consistent estimator, type 3. *R* ^2^ = model explained variance; Δ*R* ^2^ = additional variance explained by the interaction. Covariate coefficients are omitted. In the compassion fatigue models, coefficients for focal appraisals and peer support are conditional on the other interacting variable being at its mean.

Abbreviations: CI = confidence interval, SE = standard error.

The associations among perceived duty difficulty, EOLCS, and compassion fatigue are depicted in Figure 2. Perceived duty difficulty was significantly associated with EOLCS (*B* = 0.52, SE = 0.13, 95% CI [0.26, 0.78]), and EOLCS was significantly related to compassion fatigue (*B* = 5.44, SE = 1.25, 95% CI [2.97, 7.91]) after adjustment for the covariates. The EOLCS model explained 27.7% of the variance.

**FIGURE 2:**
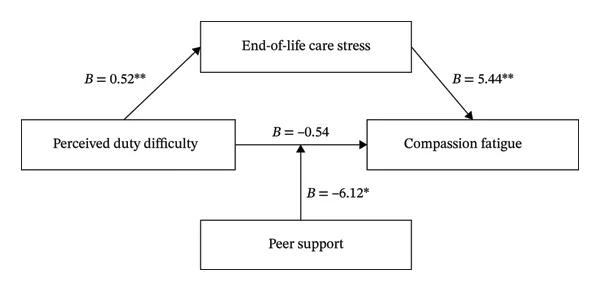
Cross‐sectional indirect association between perceived duty difficulty and compassion fatigue through end‐of‐life care stress and interaction with peer support. *Note.* Values are unstandardized regression coefficients. The coefficient on the path from perceived duty difficulty to compassion fatigue represents the conditional association at mean peer support. The interaction coefficient represents perceived duty difficulty × peer support. Perceived duty difficulty and peer support were mean‐centered. Models were adjusted for perceived duty importance and frequency, hospice work experience, and education level. ∗*p* < 0.01; ∗∗*p* < 0.001.

In the sensitivity analysis excluding perceived duty importance and frequency from adjustment, the cross‐sectional indirect association between duty difficulty and compassion fatigue through EOLCS remained positive and statistically significant (effect = 2.82, Boot SE = 0.92, 95% bootstrap CI [1.05, 4.64]). The corresponding indirect associations for perceived duty importance and frequency remained nonsignificant.

### 3.4. Interactions Between Duty Appraisals and Peer Support in Relation to Compassion Fatigue

Table 4 presents the interactions between peer support and the three duty‐appraisal dimensions in relation to compassion fatigue. After adjusting for the covariates, the interaction between perceived duty difficulty and peer support was statistically significant (*B* = −6.12, SE = 2.29, *p* = 0.009, 95% CI [−10.65, −1.59]). The outcome model explained 23.7% of the variance in compassion fatigue, with the interaction term accounting for an additional 8.2% of the variance (Δ*R* ^2^ = 0.0821). Figure 3 illustrates the conditional association between perceived duty difficulty and compassion fatigue at different levels of peer support. Simple slope analyses based on the mean‐centered model indicated that the association was positive but not statistically significant at a low level of peer support (−1 SD; *B* = 3.20, SE = 2.35, 95% CI [−1.46, 7.85]). At the mean level of peer support, the association was also not statistically significant (*B* = −0.54, SE = 1.50, 95% CI [−3.52, 2.43]). At a high level of peer support (+1 SD), the association was significantly negative (*B* = −4.28, SE = 1.70, 95% CI [−7.65, −0.91]). The interactions involving perceived duty importance and frequency were not statistically significant (*p* = 0.366 and 0.828, respectively).

**FIGURE 3:**
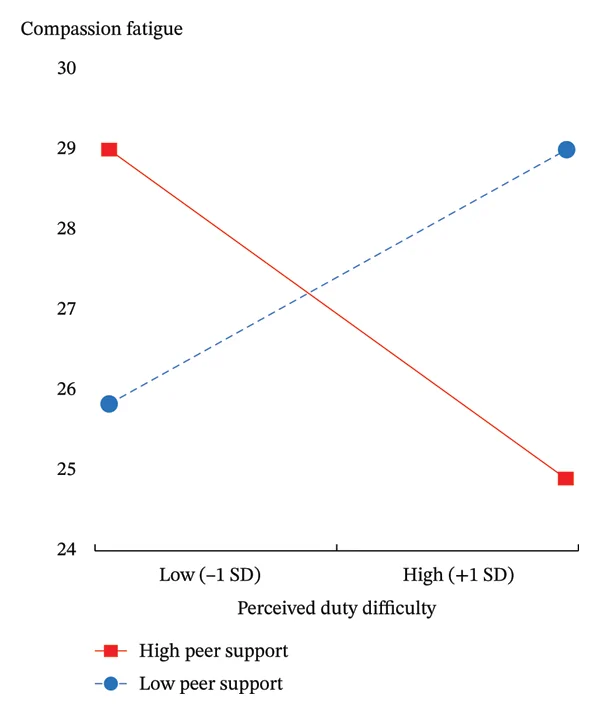
Interaction between perceived duty difficulty and peer support in relation to compassion fatigue. *Note*. Values represent adjusted predicted compassion fatigue scores, controlling for end‐of‐life care stress, education level, hospice work experience, perceived duty importance, and perceived duty frequency. Low and high duty difficulty on the horizontal axis correspond to M − 1 SD and M + 1 SD, respectively; the two lines represent low (M − 1 SD) and high (M + 1 SD) peer support. The slope was nonsignificant at low peer support (*B* = 3.20, *p* = 0.176) and significantly negative at high peer support (*B* = −4.28, *p* = 0.013). M = mean; SD = standard deviation.

The Johnson–Neyman analysis identified two regions of significance. The conditional association between perceived duty difficulty and compassion fatigue was statistically significant and positive when mean‐centered peer support was below −1.28, was not statistically significant between −1.28 and 0.39, and was statistically significant and negative when mean‐centered peer support exceeded 0.39. These findings indicate a crossover interaction, although the significant positive region contained only 3.4% of participants, compared with 29.1% in the significant negative region.

In the sensitivity analysis excluding perceived duty importance and frequency from adjustment, the interaction between duty difficulty and peer support remained significant (*B* = −6.05, HC3 SE = 2.27, *p* = 0.009, 95% CI [−10.54, −1.56]). The conditional association remained nonsignificant at low and mean peer support and significantly negative at high peer support, consistent with the primary analysis. The interactions involving perceived duty importance and frequency remained nonsignificant.

## 4. Discussion

Given the unique nature of hospice care work, in which nurses assume primary responsibility for delivering holistic care through sustained empathetic engagement, compassion fatigue among hospice nurses represents a critical concern with implications for both the quality of hospice care and nurses’ professional quality of life. This study examined the associations between nurses’ appraisals of hospice care duties (importance, frequency, and difficulty) and compassion fatigue, including cross‐sectional indirect associations through EOLCS and variation in these associations according to peer support among nurses in Korean inpatient hospice units.

Regarding the associations between nurses’ appraisals of hospice duties and compassion fatigue, perceived duty difficulty emerged as the key appraisal showing a cross‐sectional indirect association with compassion fatigue through EOLCS. Nurses who perceived their duties as more difficult reported greater EOLCS, and those reporting greater EOLCS also reported higher compassion fatigue. This pattern is consistent with previous accounts of repeated encounters with dying patients and emotionally charged interactions with families as central stressors in hospice and palliative care [31, 32] and with evidence linking EOLCS to nurses’ psychological distress [33, 34]. These findings suggest that the emotional demands of end‐of‐life care are relevant to understanding the association between perceived duty difficulty and compassion fatigue, although the temporal ordering of these relationships cannot be established.

The distinction between perceived duty difficulty and the other appraisal dimensions also warrants attention. Perceived importance concerns the value nurses assign to their duties, whereas frequency reflects how often those duties are performed; neither necessarily captures how demanding nurses find them. The findings therefore draw attention to nurses’ subjective experience of difficulty as a distinct aspect of hospice work. Previous research has linked EOLCS to heavy workloads and intense emotional demands [11], providing context for this interpretation. However, perceived difficulty should not be equated with objective workload, as it may also reflect the complexity of care and the resources nurses perceive as available to meet its demands.

Notably, the association between perceived duty difficulty and compassion fatigue differed according to the level of peer support. The observed crossover pattern does not support a straightforward buffering interpretation. Greater perceived difficulty was associated with lower compassion fatigue among nurses reporting high peer support, whereas evidence for the opposite relationship among those with low support was limited. This finding was similar whether or not the other duty‐appraisal dimensions were taken into account. One possible explanation is that nurses with strong peer support perceive difficult hospice duties as shared professional challenges [21, 31]. However, this interpretation remains speculative because the proposed mechanism was not assessed.

Previous studies have described peer support as a resource for managing work‐related distress, coping with difficult deaths, and navigating ethically complex care [21, 24, 35–37]. Although this literature provides context for interpreting the present findings, it does not establish why greater duty difficulty was associated with lower compassion fatigue at high peer support. Hospice work experience was included as a covariate, but competence, resilience, role clarity, unit climate, and team functioning were not assessed and may offer alternative explanations for the observed pattern [7, 38–40]. The unexpected inverse association may also reflect characteristics of this sample and requires replication in independent samples, preferably using longitudinal designs. It should not be interpreted as evidence that difficult duties are beneficial in supportive work environments. The content of perceived duty difficulty also warrants attention. Education and counseling received the highest difficulty rating and were also rated as highly important, whereas consultation, regulation, and collaboration were least frequently performed. These patterns may inform assessments of training and support needs, although the study did not establish whether they reflected time pressure or workload constraints.

Several limitations should be acknowledged. First, the cross‐sectional design precludes causal inference and permits only correlational interpretations of the relationships among duty appraisals, EOLCS, and compassion fatigue. Second, self‐report measures may be subject to common method bias, recall bias, and social desirability effects. Future studies using multiple data sources could help corroborate the findings. Third, compassion fatigue was operationalized as secondary traumatic stress, consistent with Figley’s conceptualization [26]; the findings therefore reflect this specific dimension and may not generalize to broader conceptualizations of compassion fatigue, including those encompassing burnout. Fourth, participants were direct‐care nurses working in Korean inpatient hospice units serving patients with terminal cancer, limiting generalizability to home‐ or community‐based hospice settings, noncancer populations, and other cultural contexts. Although nurses were recruited from multiple institutions, the absence of institutional identifiers precluded assessment of between‐institution differences and potential clustering within hospice units. Data were collected in early 2020, approximately six years before the present report; subsequent changes in hospice work environments may limit their applicability to current nursing‐management practice. Finally, power for the cross‐sectional indirect associations was not specifically established, so nonsignificant findings should be interpreted cautiously.

Despite these limitations, the study contributes to a more nuanced understanding of how hospice duty appraisals relate to compassion fatigue among nurses in Korean inpatient hospice settings. The findings underscore the importance of attending to perceived duty difficulty, particularly in the most challenging domains of practice, alongside EOLCS and peer support as relevant aspects of nurses’ work experiences. Collectively, these findings add to the evidence base for nursing management focused on supporting nurses’ well‐being and sustaining high‐quality hospice care.

## 5. Implications for Nursing Management

The findings identify perceived duty difficulty, EOLCS, and compassion fatigue, together with the peer‐support context, as areas for nursing‐management attention and further assessment in inpatient hospice settings. The duty‐specific ratings may inform local discussions of training and support needs, particularly regarding patient education and counseling. Such assessments should establish whether and how the findings apply to current conditions in individual hospice units.

Structured peer‐support opportunities, reflective debriefing after difficult end‐of‐life care experiences, and mentoring for nurses with limited hospice experience are candidate strategies for future implementation and evaluation [21, 24, 37]. Where local assessments identify relevant needs, these initiatives could incorporate discussion of difficult clinical situations and opportunities for supervised learning. Pilot initiatives should assess their acceptability, feasibility, and potential effects on EOLCS and compassion fatigue. The observed crossover interaction does not establish that strengthening peer support reduces compassion fatigue or that greater duty difficulty is beneficial in supportive environments.

Staffing arrangements, scheduling, and protected time for participation should be considered when planning such initiatives, although their effects were not examined in this study. Periodic assessment of EOLCS and compassion fatigue, alongside reviews of perceived duty difficulty and workload, could also be evaluated as an approach to identifying support needs and linking nurses with appropriate resources. Prospective evaluations are needed to determine whether these strategies provide meaningful and sustainable benefits for hospice nurses.

## 6. Conclusion

Among nurses’ appraisals of hospice duties, perceived duty difficulty showed a cross‐sectional indirect association with compassion fatigue through EOLCS. Peer support interacted with perceived duty difficulty in relation to compassion fatigue, showing a crossover pattern in which the direction of the conditional association varied across levels of peer support. The unexpected negative association at high peer support requires replication and does not demonstrate a protective effect of peer support. These findings identify EOLCS and peer support as potentially relevant factors in understanding the associations between hospice care duties and compassion fatigue. Future longitudinal and intervention studies are needed to examine these associations across diverse hospice settings and to evaluate organizational approaches addressing EOLCS and peer support.

## Author Contributions

Conceptualization, Haerim Lee and Hyun‐E Yeom; methodology, Hyun‐E Yeom and Haerim Lee; investigation, Haerim Lee; formal analysis, Haerim Lee and Hyun‐E Yeom; validation, Hyun‐E Yeom; writing–original draft preparation, Haerim Lee; writing–review and editing, Hyun‐E Yeom; supervision, Hyun‐E Yeom; project administration, Hyun‐E Yeom.

## Funding

This research received no external funding.

## Disclosure

The funder had no role in the study design; data collection, analysis, or interpretation; manuscript preparation; or the decision to submit the manuscript for publication.

## Ethics Statement

The research procedures were reviewed and approved by the Institutional Review Board of Chungnam National University (approval no. 201906‐SB‐095‐01).

## Conflicts of Interest

The authors declare no conflicts of interest.

## Supporting Information

Additional supporting information can be found online in the Supporting Information section.

## Supporting information


**Supporting Information** The supplementary file includes the completed STROBE checklist for cross‐sectional studies supporting transparency of reporting.

## Data Availability

The data that support the findings of this study are available on request from the corresponding author. The data are not publicly available due to privacy or ethical restrictions.
